# An immature inhibin‐α‐expressing subpopulation of ovarian clear cell carcinoma cells is related to an unfavorable prognosis

**DOI:** 10.1002/cam4.3801

**Published:** 2021-02-20

**Authors:** Shinya Kusumoto, Masako Kurashige, Kenji Ohshima, Shinichiro Tahara, Takahiro Matsui, Satoshi Nojima, Satoshi Hattori, Eiichi Morii

**Affiliations:** ^1^ Department of Pathology Osaka University Graduate School of Medicine Osaka Japan; ^2^ Division of Biomedical Statistics Department of Integrated Medicine Graduate School of Medicine, and Institute for Open and Transdisciplinary Research Initiatives Osaka University Osaka Japan

## Abstract

Inhibin‐α, a member of transforming growth factor‐β, is elevated in multiple tumors, but its specific roles are poorly understood. Here, we examined the feature of inhibin‐α‐expressing cells in ovarian tumors. Immunohistochemically, inhibin‐α‐expressing tumor cells were detected only in ovarian clear cell carcinoma (OCCC) among various types of ovarian tumors. By comparing the expression of inhibin‐α and Ki‐67, inhibin‐α‐expressing tumor cells were revealed to be less proliferative. When spheroids and chemoresistant cells were derived from OCCC cell lines, the expression level of inhibin‐α was elevated, and that of an immature marker, aldehyde dehydrogenase, was also elevated. In consistent with this, inhibin‐α expression was correlated with other immature markers, such as OCT3/4 and SOX2, and inversely correlated with proliferative marker MKI67 in public database on OCCC. Knockdown of inhibin‐α in OCCC cell decreased chemoresistance. Moreover, prognostic analysis with 69 surgically resected OCCC cases revealed that the increased inhibin‐α expression was an independent unfavorable prognostic factor. These findings suggested that inhibin‐α‐expressing subpopulation of OCCC tumor cells appeared to be less proliferative, immature, and angiogenic and to be related to acceleration of malignant progression.

## Graphical abstract

Inhibin‐α‐expressing subpopulation was less proliferative and upregulated in the recurrent site, spheroids and chemoresistant cells. Moreover, those cells had immature properties and high angiogenic potential, leading to unfavorable prognosis

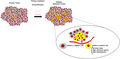

## 1 INTRODUCTION

Ovarian clear cell carcinoma (OCCC) is a rarer histological type of ovarian tumor in western countries than in Japan.^1^, ^2^, ^3^ OCCC has low sensitivity to chemotherapy.^1^, ^4^, ^5^ However, OCCC patients are still treated with conventional chemotherapies such as platinum and taxane,^6^ because effective alternative treatments have not been identified. Therefore, the prognosis of OCCC is poor, particularly in advanced stages.^7^ Recently, a subpopulation of tumor cells with immature and dormant feature has been described to be critically involved in metastatic dissemination and therapy resistance.^8^, ^9^, ^10^, ^11^, ^12^ There is currently no biomarker that can efficiently reflect such subpopulation in OCCC. Therefore, the development of novel therapy and biomarkers for them is important.

There are some biomarkers for immature subpopulations in several cancers, such as aldehyde dehydrogenase (ALDH) and CD44 ^13^, ^14^, ^15^, ^16^ and are often used to evaluate cancer cell stemness. Transforming growth factor‐β (TGF‐β) acts to promote or suppress cancer and has two‐sided effects.^17^ We previously demonstrated that Nodal, which belongs to the TGF‐β family, had an inhibitory effect on the expression of ALDH in endometrioid adenocarcinoma of uterus.^18^ In this study, we investigated the feature of subpopulation with inhibin‐α expression, which belongs to the TGF‐β family, in OCCC.

Inhibin includes inhibin‐A and inhibin‐B; the former is composed of inhibin‐α and inhibin‐βA units, and the latter is composed of inhibin‐α and inhibin‐βB units. Inhibin‐α is used as a biomarker for granulosa cell tumor. Inhibin levels are elevated in several cancers ^19^, ^20^, ^21^; however, its function has not been fully elucidated. Inhibin‐A has only been reported to promote tumor angiogenesis in ovarian cancer, but its precise function in OCCC has not been reported.^22^

Here, we presented the tumor heterogeneity in OCCC based on inhibin‐α. Inhibin‐α‐expressing cells were less proliferative and had immature feature. Inhibin‐α expression was correlated to angiogenic markers. Moreover, multivariate analysis with clinical samples revealed the presence of inhibin‐α‐expressing tumor cells to be an independent unfavorable prognostic factor of OCCC. These findings suggested that inhibin‐α‐expressing subpopulation appeared to accelerate the malignant progression of OCCC.

## 2 MATERIALS AND METHODS

### 2.1 Study design

The study was conducted as a retrospective study. After obtaining written informed consent, 99 patients with ovarian tumor who underwent surgery from 2006 to 2017 in Osaka University Hospital (Osaka, Japan) were enrolled. The study was approved by the Ethical Review Board of the Graduate School of Medicine, Osaka University (No. 15234). Resected specimens were fixed in 10% formalin and processed for paraffin embedding. The specimens were stored at room temperature in a dark room.

### 2.2 Cell lines

The human OCCC cell lines OVTOKO, RMGV, and OVISE were obtained from the Health Science Research Resources Bank of Osaka, Japan. OVTOKO and OVISE were cultured in RPMI (Nacalai Tesque) supplemented with 10% fetal bovine serum (FBS) (Biosera). RMGV was cultured in Ham‐F (Nacalai Tesque) supplemented with 10% FBS (Biosera, Nuaille, France). All cell lines were certified to be free of fungal, bacterial, and mycoplasma contaminations by the cell bank.

### 2.3 Antibodies

The antibody against inhibin‐α (R1, ab14087, Abcam, Cambridge, United Kingdom) was used for immunohistochemistry and immunofluorescence analyses (dilution at 1:200). The antibody against inhibin‐α (4A2F2, ab47720, Abcam) was used for immunoblotting (dilution at 1:500) and flow cytometry analyses (1 µg for 10^6^ cells.). The antibody against ALDH1A1 (D9Q8E, 54135, Cell Signaling Technology, Inc., Danvers) was used for immunohistochemistry (dilution at 1:2000) and immunoblotting (dilution at 1:1000) analyses. Antibody for immunohistochemistry against Ki‐67 (MIB‐1, M7240, dilution at 1:100) was obtained from Dako/Agilent Technologies, Inc. (Santa Clara). Antibody for immunoblotting against β‐actin (horseradish peroxidase [HRP] conjugate) (13E5, 5125, dilution at 1:1000) was obtained from Cell Signaling Technology, Inc. The antibody against vascular endothelial growth factor (VEGF)‐A (VG‐1, ab1316, Abcam) was used for immunohistochemistry and immunoblotting (dilution at 1:200). The antibody against VEGF‐C (9/E7, ab106512, Abcam) was used for immunohistochemistry and immunoblotting (dilution at 1:400).

### 2.4 Spheroid culture (Three‐dimensional (3D) culture)

Cells were applied to a pluriStrainer (pluriSelect Life Science UG & Co. KG, Leipzig). The collected cells were seeded in ultra‐low attachment culture dishes (Corning Inc.) and cultured for 9 days in Cancer Stem Cell Media Premium (ProMab Biotechnologies, Richmond). Spheroids were counted in five random fields per well at high magnification.

### 2.5 Cell block preparation

Cells were rinsed with phosphate buffered saline (PBS), fixed with 10% formaldehyde for 10 min, and dehydrated with 100% ethanol over 3–12 h. The pellet obtained thereby was placed in the tissue cassette and dehydrated with 100% ethanol for 1 h. After the dehydration, cassette was soaked in xylene for more than 1 h. Routine processing and embedding in paraffin were done.

### 2.6 Generation of chemoresistant cells

Cells (1 × 10^4^) were seeded in each well of 96‐well plate 12–24 h prior to the incubation with carboplatin (CBDCA, Merck KGaA). Cells were incubated with CBDCA for 14–21 days. Cells were rinsed with PBS and incubated with culture solution until new colonies appeared. New colonies were seeded in a 96‐well plate and incubated with CBDCA. This process was repeated at least four times. Chemoresistant cell of RMGV and OVTOKO were named as RMGV‐R and OVTOKO‐R, respectively.

### 2.7 Immunohistochemistry

Immunohistochemical staining was conducted by the Dako Autostainer Link 48 (Dako/Agilent Technologies, Inc.) according to the manufacturer's instructions. We determined the cytosol staining of cancer glands as positive. Negative inhibin‐α expression was designated when cytosol staining was not present in any cancer cells. The intensity of signal was evaluated to scores 1–3 as described in Figure 4F. H‐scores were assigned using the following formula: [1× (% cells of score 1) +2× (% cells of score 2) +3× (% cells of score 3)]. Two pathologists (S.T. and T.M) assessed the specimens independently.

### 2.8 Immunofluorescence

Cells were rinsed with PBS, fixed with 4% paraformaldehyde in PBS for 10 min and permeabilized and blocked with 4% bovine serum albumin, 0.5% Triton X‐100 and 0.04% sodium azide in PBS for 30 min. After COVERGLASS 15 mm (MATSUNAMI) was placed in 24‐well dish culture plates (Greiner Bio‐One, Frickenhausen, Germany), Matrigel^®^ Matrix (Corning Inc.) was applied on it. Cells were applied in Matrigel and incubated overnight at 4℃ with primary antibody. The dish was washed with PBS and incubated for 1 h with Alexa Fluor‐conjugated secondary antibody (Thermo Fisher Scientific). Next, the dish was washed with PBS and counterstained with 4′,6‐diamidino‐2‐phenylindole (DAPI) (Nacalai Tesque). Multiple immunostainings of surgical specimens were performed using the Tyramide Signal Amplification Kit (Thermo Fisher Scientific), according to the manufacturer's protocol. Fluorescence signals were visualized using the Zeiss LSM 710 confocal microscope and ZEN microscope software (Carl Zeiss).

### 2.9 Drug sensitivity assay (WST‐1 assay)

1 × 10^4^ cells were seeded into 96‐well culture plates and incubated for 12–24 h at 37°C with 5% CO_2_. We subsequently added CBDCA (0, 10, 25, 50, 100, 150, or 200 μM). After 72 h incubation, cell viability was assayed by the Premix WST‐1 Cell Proliferation Assay System (Takara Bio Inc.) according to the manufacturer's instructions. The absorbance (450 and 650 nm) was measured using SH‐9000 Lab microplate reader (Hitachi) after 30–60 min incubation.

### 2.10 Cell cycle assay

A total of 1 × 10^6^ cells were washed three times and resuspended in cold PBS. Resuspended cells were added into the tube containing 1 ml of ice cold 70% ethanol while vortexing at medium speed. The tubes were frozen at −20°C for 3–24 h prior to staining. Subsequently, cells were washed and treated with 200 μl of Muse™ Cell Cycle reagent (Millipore Corp.) according to the manufacturer's protocol. After 30 min of incubation at room temperature in the dark, cell suspension samples were transferred into 1.5‐ml microcentrifuge tubes and analyzed using the Muse™ Cell Analyzer (Millipore Corp.).

### 2.11 Flow cytometry

The ALDEFLUOR kit (STEM CELL Technologies) was used to isolate the population with a high ALDH enzymatic activity, according to the manufacturer's instructions. Flow cytometry sorting was conducted using a Cell Sorter SH800ZDP (SONY). To evaluate inhibin‐α‐high populations, cells were incubated with anti‐inhibin‐α antibody for 60 min and then with Alexa 488‐labeled donkey anti‐goat IgG (Invitrogen, Molecular Probes) for 30 min. We set the gating to collect inhibin‐α‐low and inhibin‐α‐high cells using the cells incubated with Alexa 488‐labeled donkey anti‐goat IgG only as the negative control.

### 2.12 Immunoblotting

Cells were rinsed three times with ice‐cold PBS and lysed in ice‐cold lysis buffer (1% Nonidet P‐40, 10‐mM Tris‐HCl, 200‐mM NaCl, 1‐mM ethylenediaminetetraacetic acid [EDTA]) containing EDTA‐free complete protease inhibitor cocktail (Merck KGaA) and PhosSTOP (Merck KGaA) for 15 min at 4°C. The soluble fractions from cell lysates were isolated by centrifugation at 21,900 G for 5 min at 4°C. Protein concentration was determined using the supernatant and the BCA protein assay kit (Thermo Fisher Scientific). Electrophoresis was carried out in 5%–20% gradient SDS‐polyacrylamide gels (ATTO), and proteins were transferred to PVDF membranes (Merck KGaA). The membrane was blocked with 4% non‐fat dry milk for 20 min. Bound antibodies were detected with HRP‐conjugated antibodies specific for mouse or rabbit IgG (H + L chain) (MBL, dilution at 1:1000) and with Western Lightning Plus‐ECL (PerkinElmer, Inc.). LAS‐4000 Image Analyzer (GE Healthcare) was used for the detection of antibody reaction. The expression of β‐actin was used as a loading control. Coomassie Brilliant Blue (CBB) (APRO SCIENCE) staining was also used for loading control.

### 2.13 siRNA‐mediated silencing of inhibin‐α in OCCC cell line

OVISE cell seeded at 70% confluence were transfected with an inhibin‐α‐targeting siRNA (Silencer Select s51538, s51539; Thermo Fisher Scientific) or non‐targeting control siRNA (AM4611; Thermo Fisher Scientific), using Lipofectamine RNAiMAX reagent (Thermo Fisher Scientific) at a final concentration of 50 nmol/L. Sequences of the inhibin‐α‐targeting siRNAs were 5ʹ‐CCUUCUCACGCAGCACUGUTT‐3ʹ (s194559), 5ʹ‐UGCCCAACCUUCUCACGCATT‐3ʹ (s194560), and 5ʹ‐CUCUUUCAAGUAUGAGACATT‐3ʹ (s223941).

### 2.14 Statistical analysis

In vitro experiments were performed at least three times. The data are presented as means ±standard error of the mean (SEM) of independent experiments. The overall survival (OS) was defined as the duration from the date of primary treatment until the date of death due to any cause. The disease‐free survival (DFS) was defined as the duration from the date of primary treatment until the date when progressive disease was observed. We analyzed both the OS and the DFS as follow. Association between expression of inhibin‐α and patients’ prognosis was examined by comparing the survival curves of the inhibin‐α‐positive and inhibin‐α‐negative groups, which were defined according to whether the tumor epithelium was stained with inhibin‐α even a little or not, with the Kaplan–Meier method, the logrank test and the Cox proportional hazards model. In the analyses of the OS, subjects who did not die were regarded as censored at the date of the last contact. For the DFS, subjects who did not have progressive disease were censored at the last date of the last contact if no progressive disease was confirmed with computer tomography (CT), magnetic resonance imaging, or positron emission tomography‐CT. In addition, we examined the association adjusting for potential confounding factors. Among 69 subjects in our clinical dataset, only nine subjects died and 17 experienced progressive disease. Since the number of events were very small, we adjusted only the stage in the multivariate Cox regression, which seemed the most important confounding factor. To address potential influence of multiple confounding factors, we applied the inverse probability of treatment weighted (IPW) method by using the propensity score. In estimating the propensity score, the outcome was positivity of the expression of inhibin‐α. Then the small number of events was not problematic in stable estimation of the propensity score, which was estimated with the logistic regression model with age, stage (1–2 vs. 3–4), peritoneal cytology, and operation. Statistical analyses were performed with JMP Pro 14 / 15, SAS version 9.4 (SAS Institute Inc.) and IPWsurvival function in R version 3.6 (R core tear; https://www.R‐project.org/).

## 3 RESULTS

### 3.1 Inhibin‐α expression in ovarian cancer

**FIGURE 1:**
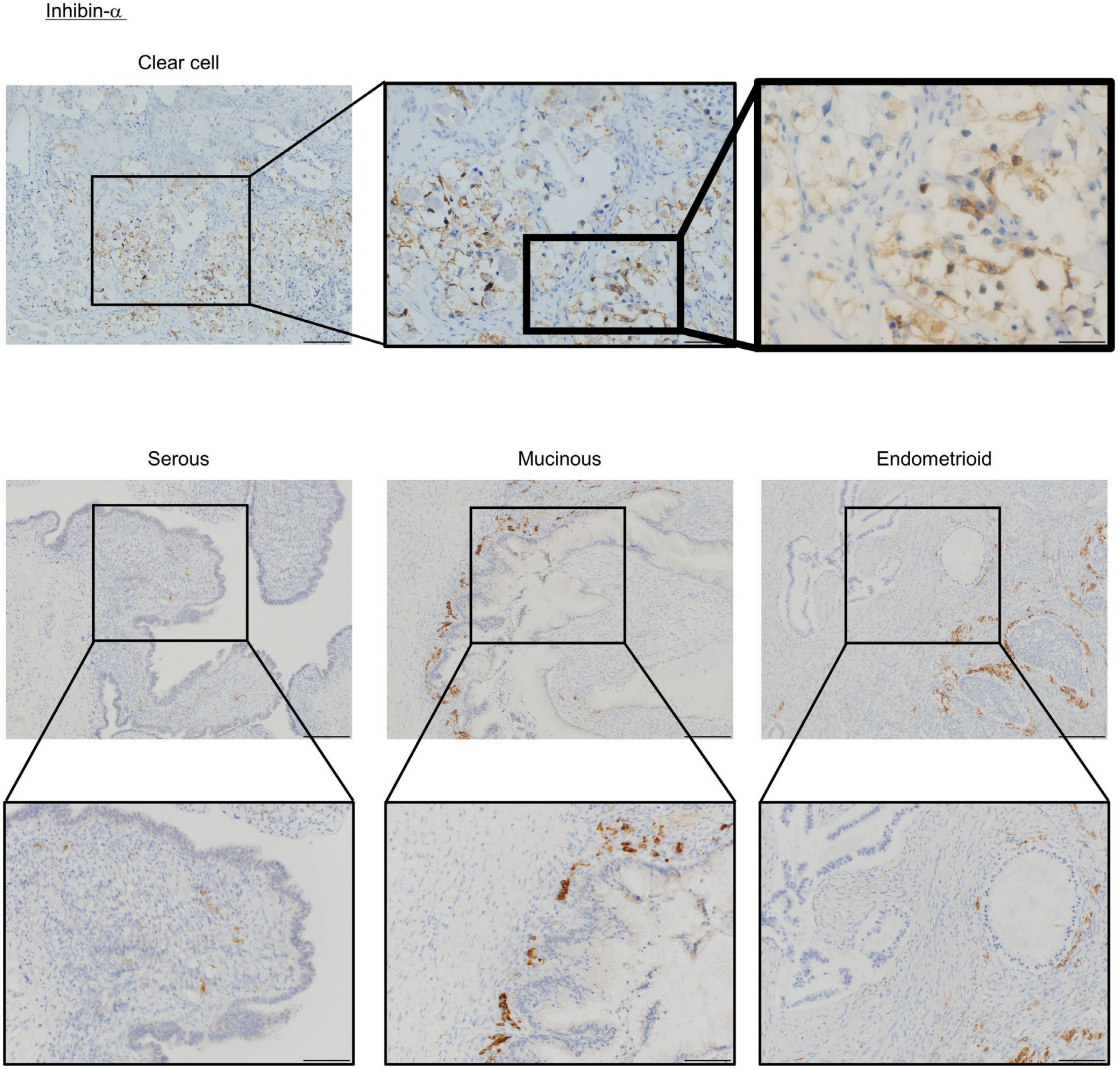
Inhibin‐α expression pattern in clinical specimens derived from human ovarian tumor tissues. Tissues were stained with mouse anti‐inhibin‐α. Scale bars (clear cell): 200 μm, 100 μm, 50 μm. Scale bars (serous, mucinous, endometrioid): 200 μm, 100 μm

We first evaluated inhibin‐α expression in ovarian cancer. Ovarian cancer tissue contained four subtypes: serous (*N* = 11), mucinous (*N* = 12), endometrioid (*N* = 7), and OCCC (*N* = 11). In three subtypes other than OCCC, inhibin‐α expression was detected only in tumor stromal cells, whereas in OCCC, inhibin‐α localization was detected in both tumor epithelial and stromal cells (Figure 1) (Tables 1 and 2).

**TABLE 1:** Inhibin‐α expression in tumor tromal cell (Fisher's exact test)

Tumor stromal cell	Non‐clear cell	Clear cell
Positive	25	5
Negative	5	6
		*p* = 0.0412

**TABLE 2:** Inhibin‐α expression in tumor epithelial cell (Fisher's exact test)

Tumor epithelial cell	Non‐clear cell	Clear cell
Positive	0	6
Negative	30	5
		*p* = 0.0001

### 3.2 Less proliferation feature of inhibin‐α‐positive cells

**FIGURE 2:**
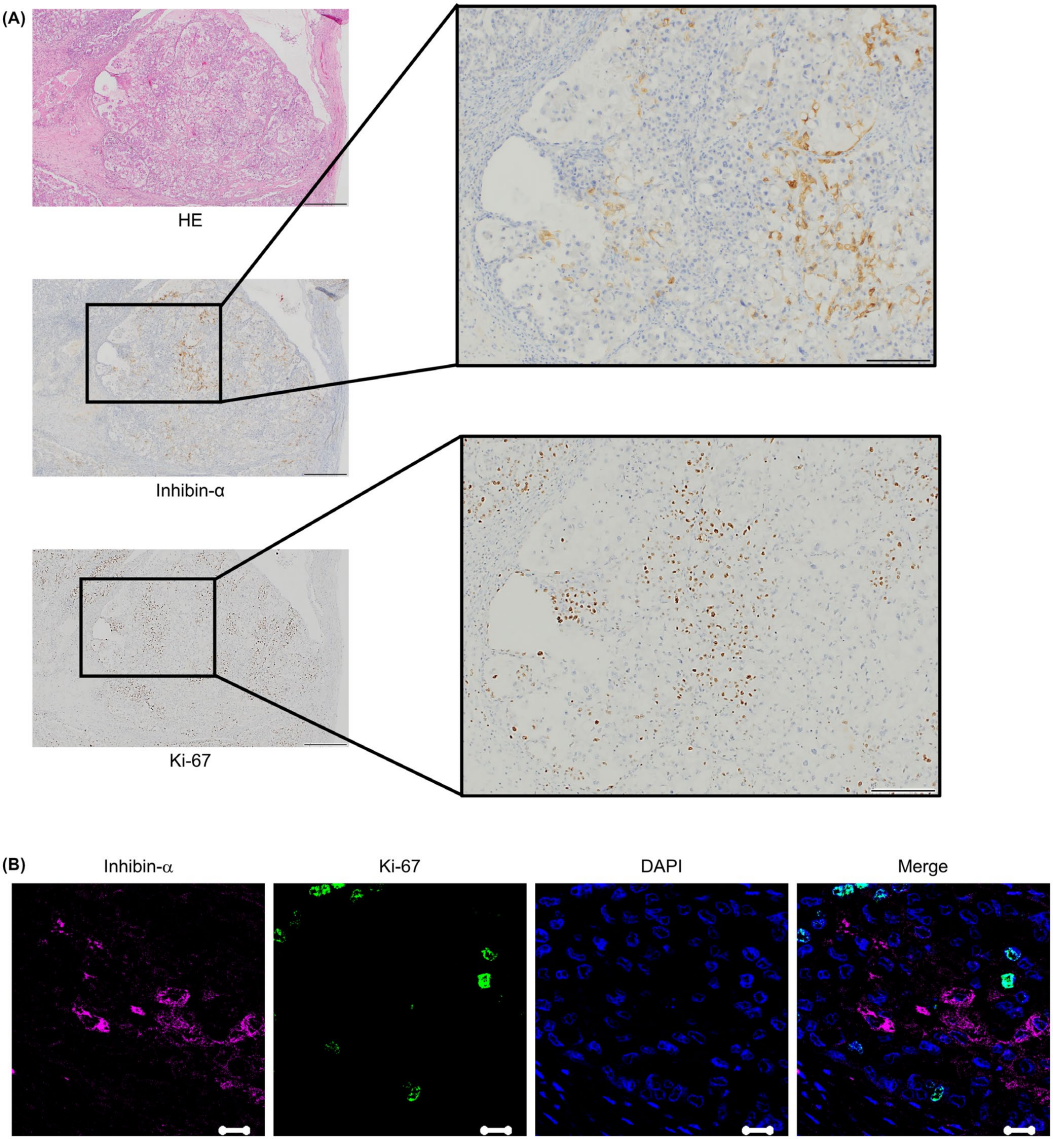
Inhibin‐α‐positive cells were less proliferative. (A) OCCC tissue sections were stained with anti‐inhibin‐α and anti‐Ki‐67 antibodies. Scale bars: 500 μm (left), 200 μm (right). (B) Representative images of immunofluorescence double staining (anti‐inhibin‐α and anti‐Ki‐67 antibodies). Scale bars: 20 μm

The expression of inhibin‐α and Ki‐67 in the surgical specimen of OCCC was immunohistochemically examined. As shown in Figure 2A, Ki‐67 was negative in almost all of cells with inhibin‐α positivity. In the experiment of the immunofluorescence double staining, we also confirmed that inhibin‐α‐positive cells were negative for Ki‐67 (Figure 2B).

### 3.3 Elevated inhibin‐α expression in spheroids

**FIGURE 3:**
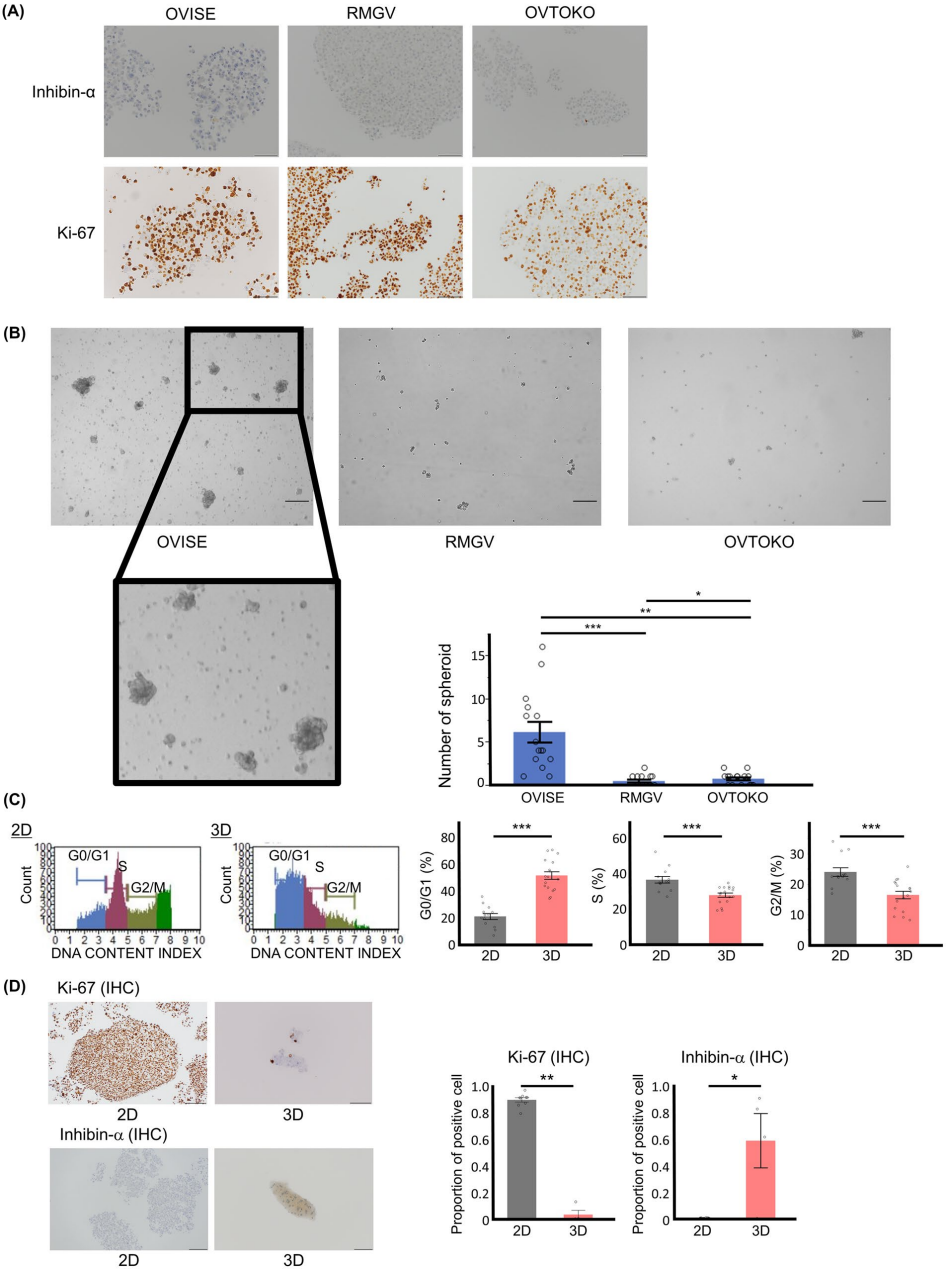
OCCC spheroids showed high inhibin‐α expression. (A) Representative images of immunohistochemistry of inhibin‐α and Ki‐67 using OCCC cell lines (OVISE, RMGV, OVTOKO). Scale bars: 200 μm. (B) Representative images of OCCC cell lines in 3D culture. Scale bars: 200 μm. (*p < 0.05, **p < 0.01, ***p < 0.001 by the Steel–Dwass nonparametric multiple comparison test). (C) Quantitation of cell cycle phases in 2D and 3D cells. (***p < 0.001 by the Wilcoxon signed‐rank test) (D) Cell blocks were stained with anti‐inhibin‐α and anti‐Ki‐67 antibodies. Scale bars: 200 μm (2D, Ki‐67), 100 μm (3D, Ki‐67). Scale bars: 100 μm (2D and 3D, inhibin‐α). (*p < 0.05, **p < 0.01 by the Wilcoxon signed‐rank test)

To confirm the relation between less proliferative cells and inhibin‐α‐positive cells, we created cell blocks from OCCC cell lines (OVISE, RMGV, OVTOKO) cultured in 2D situation. Almost all of cells were Ki‐67 positive and inhibin‐α negative (Figure 3A). Then, we conducted spheroid 3D culture. Because significant number of spheroids were observed in OVISE but not in other two RMGV and OVTOKO cell lines (Figure 3B), the following spheroid 3D experiment was done on OVISE. First, we compared the cell cycle between 2D and 3D cultures. Proportion of G1/G0 cells was higher and that of S or G2/M cells was lower in spheroid 3D culture than in 2D culture (Figure 3C). In consistent with these, the proportion of Ki‐67 positive cells was lower and the proportion of inhibin‐α‐positive cells was higher in spheroids than in 2D culture cells (Figure 3D).

### 3.4 Immature properties of inhibin‐α‐positive cells

**FIGURE 4:**
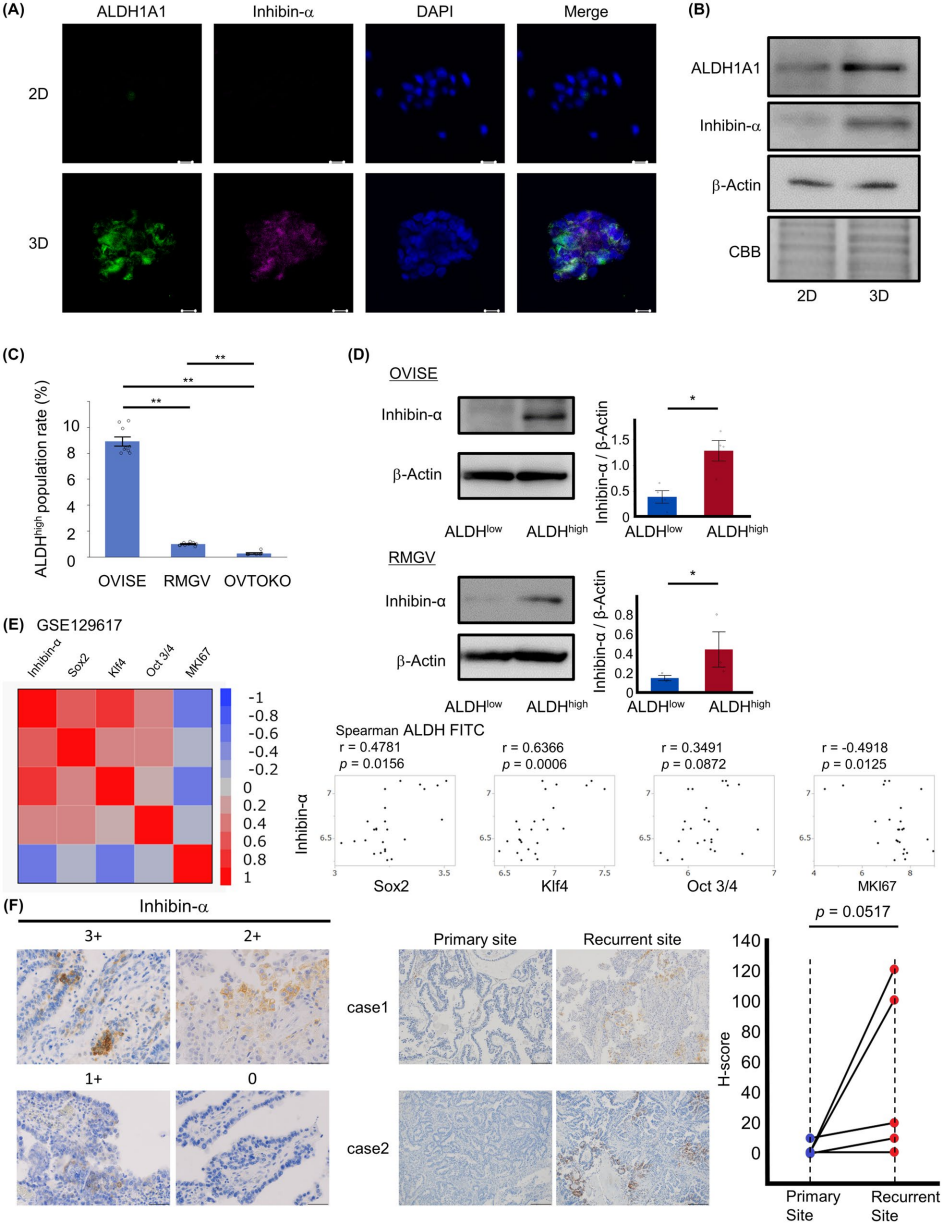
Inhibin‐α‐expressing cells showed immature property. (A) Representative images of immunofluorescence double staining (anti‐inhibin‐α and anti‐ALDH1A1 antibodies). Scale bars: 20 μm. (B) Representative immunoblotting of ALDH1A1 and inhibin‐α in 2D and 3D cells. β‐actin and CBB staining were used for loading controls. (C) Proportion of ALDH^high^ cells according to the ALDEFLUOR assay. Data are shown as mean ±SE from at least three independent experiments (**p < 0.01 by the Steel–Dwass nonparametric multiple comparison test). (D) Representative immunoblotting of inhibin‐α in sorted ALDH^high^ and ALDH^low^ cells. Inhibin‐α: β‐Actin ratios were calculated based on band intensities measured with densitometry. Data are shown as mean ±SE from at least three independent experiments (*p < 0.05 by the Wilcoxon signed‐rank test). (E) The correlation between inhibin‐α, immature stemness‐related genes, and proliferative MKI67 gene in the GSE129617. (F) Representative images of immunohistochemistry of inhibin‐α using OCCC tissues with high (score 3), intermediate (score 2), low (score 1), or null (score 0) signal intensity, which were used for calculating H‐scores. Scale bars: 50 μm. There were five recurrent cases diagnosed pathologically. OCCC tissue sections at the time of first occurrence and recurrence were stained with anti‐inhibin‐α. Scale bars: 200 μm. (*p* = 0.0517 by the Wilcoxon signed‐rank test)

In the immunofluorescence double staining, inhibin‐α and the immature marker ALDH1A1 were co‐expressed more clearly in spheroids (Figure 4A). The immunoblotting also showed inhibin‐α and ALDH1A1 were expressed more strongly in spheroids than in 2D culture cells (Figure 4B). To validate these results, we conducted immunoblotting of inhibin‐α in ALDH‐high and ALDH‐low populations of OCCC cell lines which were sorted by ALDEFLUOR assay. Of the three cell lines, we selected two (OVISE, RMGV) cell lines for ALDEFLUOR assay, because ALDH expression was hardly detected in OVTOKO (Figure 4C). In both cell lines, the ALDH‐high population showed higher levels of inhibin‐α than the ALDH‐low population (Figure 4D). In publicly available microarray datasets, GSE129617, which consisted of 25 OCCC patients, inhibin‐α was positively correlated with stemness genes (Sox2, Klf4, Oct 3/4) and negatively correlated with proliferation‐related gene MKI67 (Figure 4E). It has been reported that less proliferative cells were increased in the metastasized lesion of tumor cells.^23^, ^24^ In fact, H‐score of inhibin‐α tended to be higher in recurrent sites than in primary sites of five pathologically diagnosed recurrent cases (Figure 4F).

### 3.5 Elevated expression of inhibin‐α in chemoresistant cells

**FIGURE 5:**
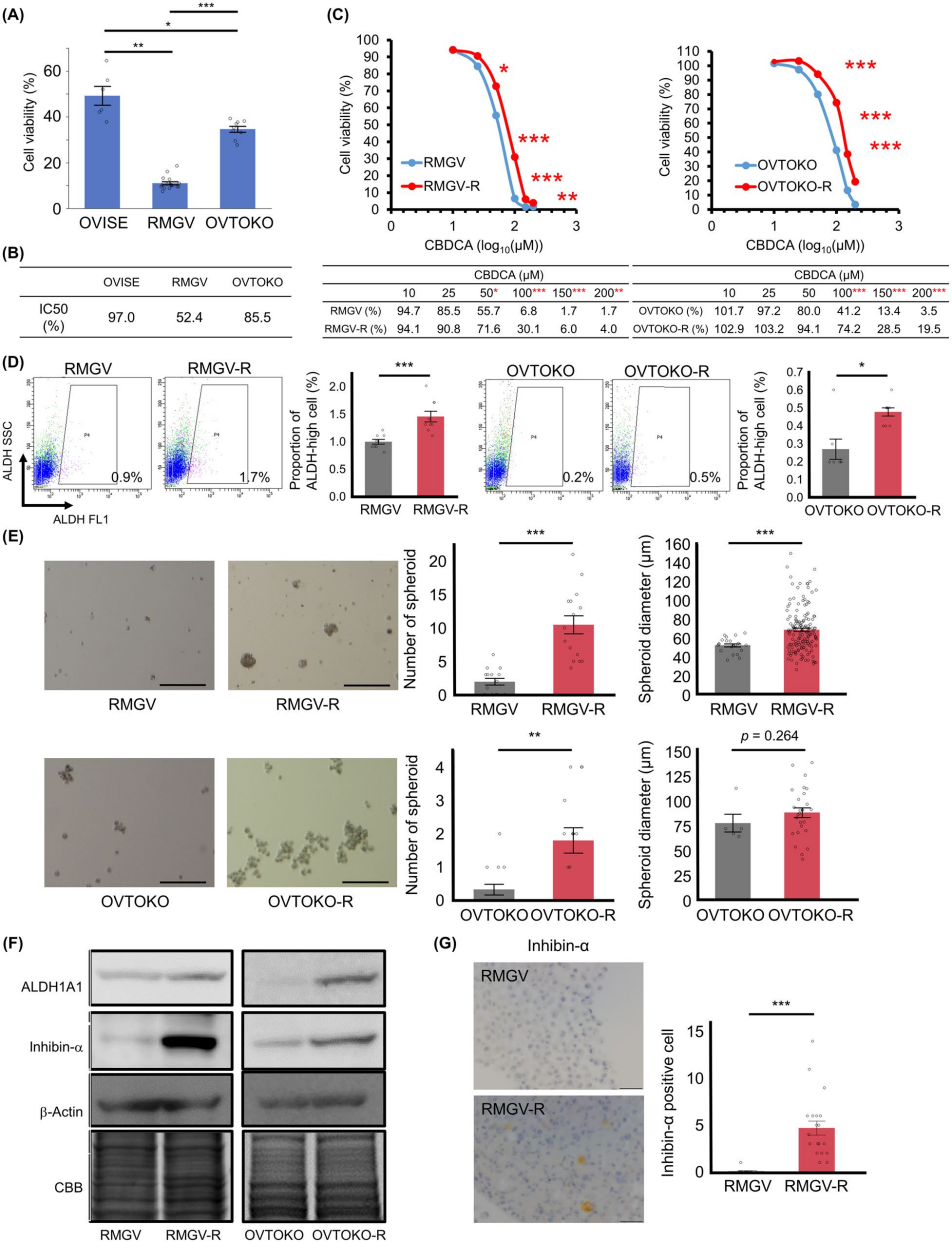
Chemoresistant cells showed enriched stemness properties and high inhibin‐α expression. (A) Cell viability (CV) in OCCC cell lines that were treated with CBDCA (Dose: 100 μM). Data were shown as mean ±SE from at least three independent experiments (*p < 0.05, **p < 0.01, ***p < 0.001 by the Steel–Dwass nonparametric multiple comparison test). (B) Half maximal Inhibitory concentration for CBDCA was assessed for each cell line. (C) Representative dose response curves of RMGV, RMGV‐R, OVTOKO, and OVTOKO‐R that were treated with CBDCA were shown (upper panel). Corresponding CV values were shown (lower panel). Data were shown as mean ±SE from at least three independent experiments (*p < 0.05, **p < 0.01, ***p < 0.001 by the Wilcoxon signed‐rank test). (D) ALDEFLUOR assay was performed on RMGV, RMGV‐R, OVTOKO, and OVTOKO‐R. Data were shown as mean ±SE from at least three independent experiments (*p < 0.05, ***p < 0.001 by the Wilcoxon signed‐rank test). (E) Representative images of spheroids in RMGV, RMGV‐R, OVTOKO, and OVTOKO‐R. Spheroids were counted in five random fields per well. The diameters of all counted spheroids were measured. Scale bars: 200 μm. Data were shown as mean ±SE from at least three independent experiments (**p < 0.01, ***p < 0.001 by the Wilcoxon signed‐rank test). (F) Immunoblotting of ALDH1A1 and inhibin‐α. β‐Actin and CBB staining were used for loading controls. (G) Representative images of cell blocks (RMGV and RMGV‐R). They were stained with anti‐inhibin‐α antibody. Scale bars: 50 μm. Data were shown as mean ±SE from at least three independent experiments (***p < 0.001 by the Wilcoxon signed‐rank test)

Chemotherapy has been associated with an increase of immature subpopulation.^25^, ^26^, ^27^, ^28^, ^29^ Therefore, we generated chemoresistant cells to confirm the elevated inhibin‐α expression. We used CBDCA which is one of the most clinically used anticancer drugs. We examined the susceptibility of three cell lines to CBDCA and calculated the IC50 to CBDCA. We selected two (RMGV, OVTOKO) cell lines for further evaluation because of the lower IC50 value (Figure 5A,B). As expected, chemoresistant cells (RMGV‐R and OVTOKO‐R) were more resistant to CBDCA than parent cells (Figure 5C) To validate upregulated stemness in chemoresistant cells, we conducted ALDEFLUOR assay and spheroid culture. Both RMGV‐R and OVTOKO‐R cells showed abundant ALDH‐high population and spheroids as compared to parent cells (Figure 5D,E). In the immunoblotting, inhibin‐α, as well as ALDH1A1, were expressed more strongly in chemoresistant cells than in parent cells (Figure 5F). We created cell blocks of RMGV and RMGV‐R and confirmed the expression of inhibin‐α by immunohistochemistry. Proportion of inhibin‐α‐positive cells in RMGV‐R was higher than in RMGV (Figure 5G).

### 3.6 Silencing of inhibin‐α reduced chemoresistance

**FIGURE 6:**
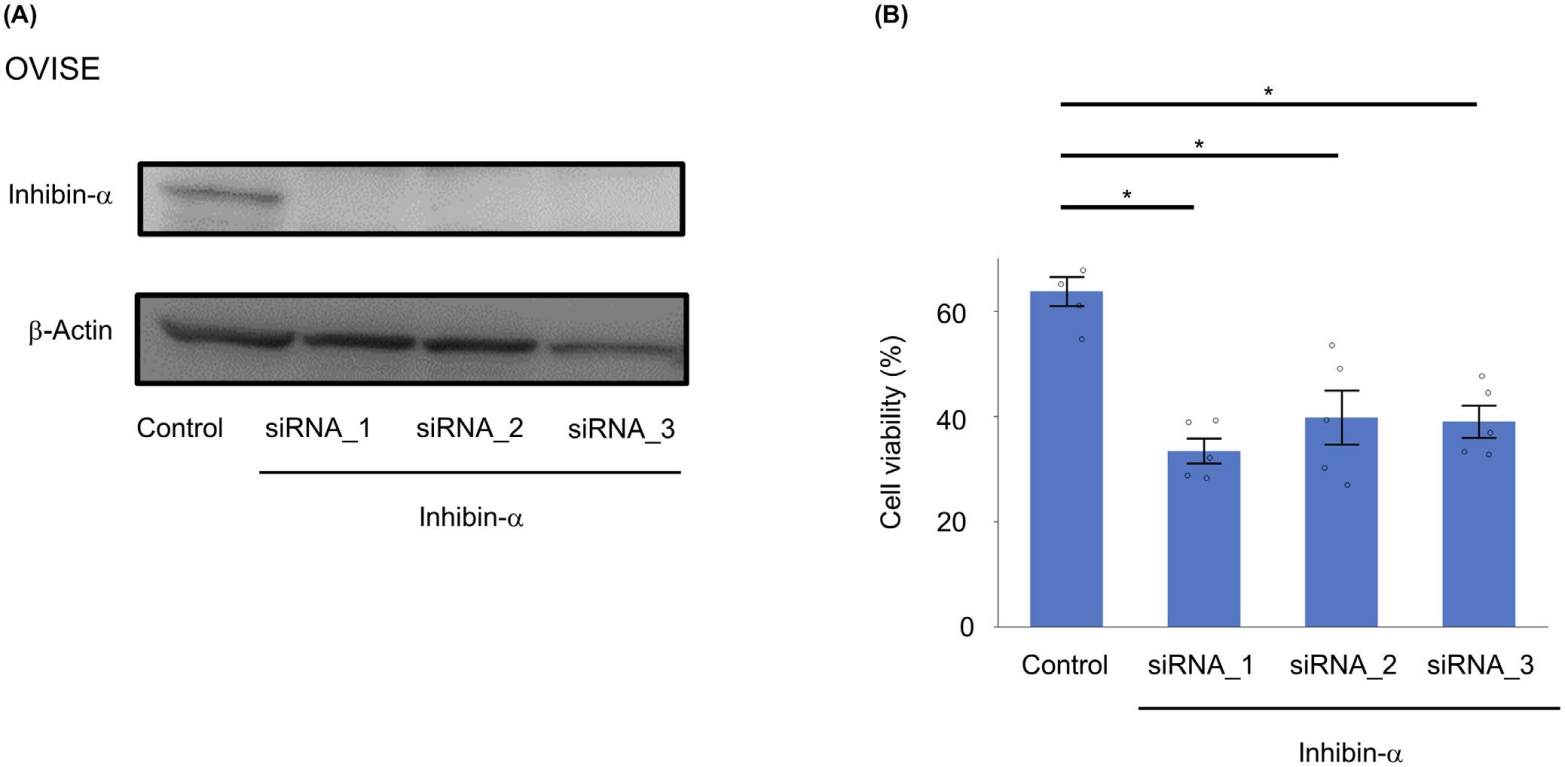
Inhibin‐α knockdown decreased chemoresistance. (A) Representative immunoblotting of inhibin‐α in OVISE cell transfected with three individual siRNA duplexes specific for inhibin‐α (si inhibin‐α _1, _2, and _3) or nontargeting control siRNA (Control). (B) CV in OVISE that were treated with CBDCA (Dose: 200 μM) Data were shown as mean ±SE from at least three independent experiments (*p < 0.05 by the Wilcoxon signed‐rank test)

To examine the mechanism by which inhibin‐α is involved in the chemoresistance of OCCC, we conducted in vitro functional assays of siRNA‐mediated inhibin‐α knockdown using OCCC cell line (OVISE). First, we transfected OVISE cell with three individual siRNA duplexes specific for inhibin‐α (si inhibin‐α _1, _2, and _3) or a non‐targeting control siRNA (Control) and confirmed the decrease in inhibin‐α protein expression in inhibin‐α knockdown cells (Figure 6A). Then, we compared the chemoresistance of inhibin‐α knockdown and control cells by WST‐1 assay. Knockdown of inhibin‐α resulted in decreased chemoresistance (Figure 6B).

### 3.7 High angiogenic potential in inhibin‐α‐positive cells

**FIGURE 7:**
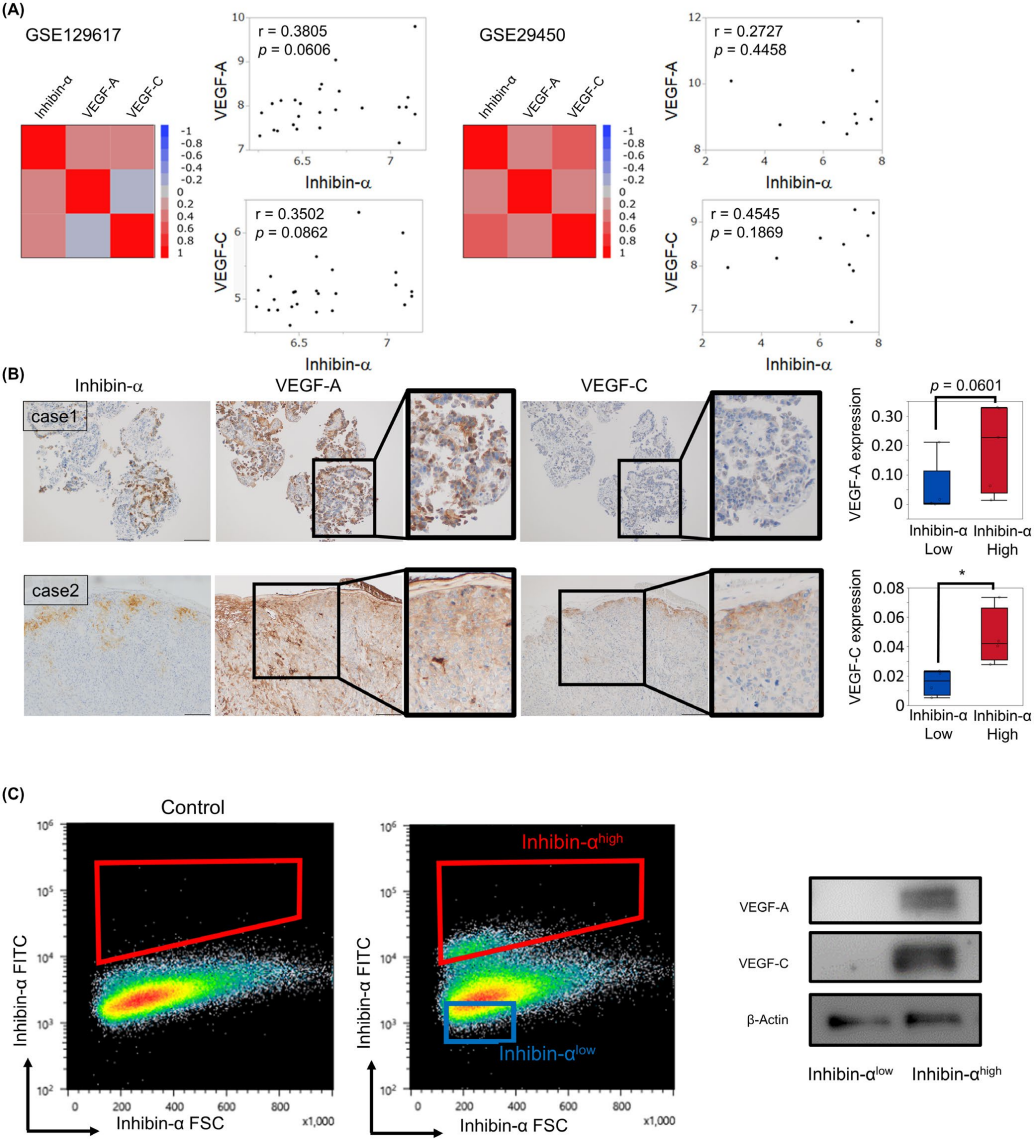
Inhibin‐α‐positive cells had high angiogenic potential. (A) The correlation between inhibin‐α, VEGF‐A, and VEGF‐C in the GSE129617 and GSE29450. (B) Representative image of immunohistochemistry for inhibin‐α, VEGF‐A, and VEGF‐C using OCCC tissues. VEGF‐A and VEGF‐C protein level were statistically analyzed by comparing H‐scores calculated in the inhibin‐α‐positive and inhibin‐α‐negative area from three cases (*p < 0.05 by the Wilcoxon signed‐rank test). (C) Representative flow cytometry gating strategy for the isolation of inhibin‐α‐high cells and inhibin‐α‐low cells was shown. Representative immunoblotting of VEGF‐A and VEGF‐C in sorted inhibin‐α‐high and inhibin‐α‐low cells was also shown

We examined characteristics of inhibin‐α‐positive cells. In the publicly available microarray dataset GSE129617 and GSE29450, which consisted of 10 OCCC patients, inhibin‐α was positively correlated with VEGF‐A and VEGF‐C (Figure 7A). As shown in Figure 7B, inhibin‐α‐positive cells showed higher VEGF‐A and VEGF‐C expression than inhibin‐α‐negative cells. To validate these results, we divided cells into inhibin‐α‐high and inhibin‐α‐low populations and conducted immunoblotting. The expression levels of VEGF‐A and VEGF‐C were higher in inhibin‐α‐high population than in inhibin‐α‐low population (Figure 7C).

### 3.8 Unfavorable prognosis in clinical cases with inhibin‐α‐positive tumor cells

**FIGURE 8:**
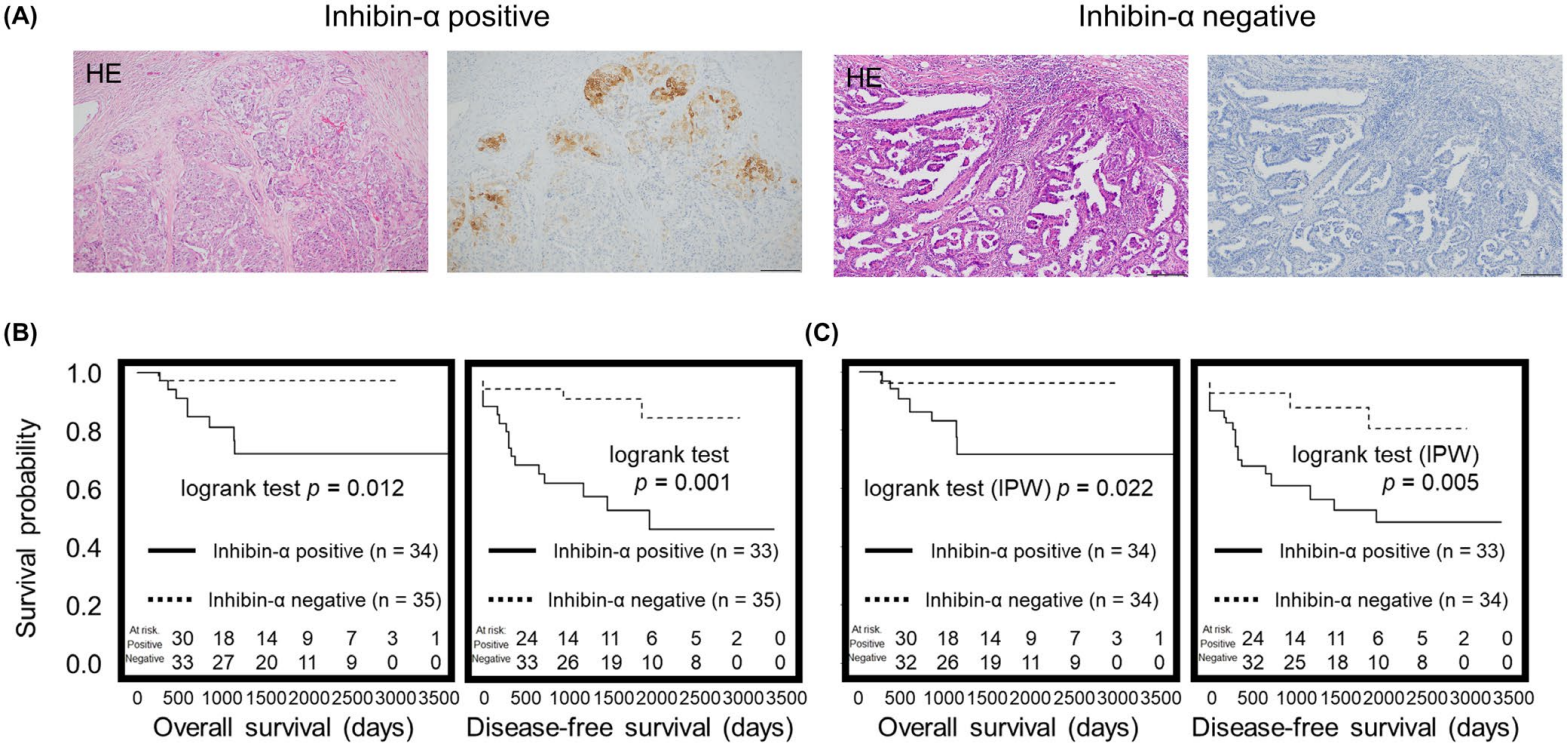
Evaluation of the OS and DFS according to inhibin‐α‐positive or inhibin‐α‐negative cases. (A) Representative images of immunohistochemistry for inhibin‐α. (B) The Kaplan–Meier plots showed substantial separations of the survival curves for OS and DFS; for the OS, *p* = 0.012, and for the DFS, *p* = 0.001 (by the log‐rank test). (C) The IPW analysis with the propensity score showed substantial separations of the survival curves for the OS and the DFS; for the OS, *p* = 0.022, and for the DFS, *p* = 0.005 (by the log‐rank test)

Patient clinicopathological information was summarized in Table 3. We divided enrolled 69 patients into two groups. When the tumor epithelium was stained with inhibin‐α even a little, the case was set as inhibin‐α‐positive group, whereas cases with no inhibin‐α expression were set as inhibin‐α‐negative group (Figure 8A). Among 69 cases of OCCC, 35 cases (51%) were inhibin‐α positive and 34 cases (49%) were inhibin‐α negative. We compared the clinicopathological features (age, stage, lymph node metastasis, peritoneal cytology, operation) of these two groups. Although no statistically significant differences were detected in these parameters, lymph node metastasis and peritoneal cytology positive cases tended to be higher in the inhibin‐α‐positive group (Table 4). Patients of the inhibin‐α‐positive group showed a significantly reduced OS and DFS rate compared with those of the inhibin‐α‐negative group (Figure 8B). One case in the inhibin‐α‐positive group was excluded from the analysis of DFS rate because of unclear recurrence date. One case in the inhibin‐α‐negative group was excluded from the analysis of OS rate because peritoneal cytology was not performed. In the inhibin‐α‐positive and inhibin‐α‐negative groups, eight cases and one case died, and 14 and three cases experienced progressive disease, respectively. Although the number of events were small, the Kaplan–Meier plots showed substantial separations of the survival curves for the OS and the DFS (Figure 8B); for the OS, *p* = 0.012 and the hazard ratio (HR) was 9.06 (95% confidence interval (CI): 1.31–72.56), and for the DFS, *p* = 0.001 and HR was 6.02 (CI: 1.73–20.97). Univariate analysis showed that stage and operation were significantly associated with OS and stage and peritoneal cytology with DFS (Tables 5 and 6). From these results, we adjusted stage in multivariate analysis. The Cox proportional hazards model showed that inhibin‐α was significantly associated with prognosis (*p* = 0.0086, HR =9.03 [CI: 1.63–168.61] for the OS and *p* = 0.0011 and HR =6.02 [CI: 1.95–26.26] for the DFS) even after adjusting for the stage (Figure 8C). In addition, we carried out IPW analysis, in which confounding factors were adjusted and concordance‐index was 0.640, showing that the adjustment of confounding factors were meaningful. The IPW analysis with the propensity score suggested the significant effects of the inhibin‐α on patients’ prognosis.

**TABLE 3:** Clinicopathological features in 69 patients with ovarian clear cell carcinoma

Clinicopathological parameters		
Characteristics		Number
Age	>60	18/69 (26%)
	≦60	51/69 (74%)
Stage	Ⅰ	48/69 (70%)
	Ⅱ	8/69 (12%)
	Ⅲ	11/69 (16%)
	Ⅳ	2/69 (3%)
Lymph node metastasis	Positive	11/69 (16%)
	Negative	57/69 (83%)
	Uncertain	1/69 (1%)
Peritoneal cytology	Positive	27/69 (39%)
	Negative	41/69 (59%)
	Uncertain	1/69 (1%)
Operation	Complete	61/69 (88%)
	Non‐complete	8/69 (12%)
Inhibin‐α	Positive	34/69 (49%)
	Negative	35/69 (51%)

**TABLE 4:** Clinicopathological features between inhibin‐α positive and negative group (Fisher's exact test)

Clinicopathological parameteres				
		Inhibin‐α in tumor cells		
		Positive	Negative	
Characteristics				*p*‐value
Age	>60	8/34 (24%)	10/35 (29%)	0.7851
	≦60	26/34 (76%)	25/35 (71%)	
Stage (1–2 vs 3–4)	Ⅰ	20/34 (59%)	28/35 (80%)	0.3707
	Ⅱ	6/34 (18%)	2/35 (6%)	
	Ⅲ	7/34 (21%)	4/35 (11%)	
	Ⅳ	1/34 (3%)	1/35 (3%)	
Lymph node metastasis	Positive	8/34 (24%)	3/34 (9%)	0.1863
	Negative	26/34 (76%)	31/34 (91%)	
Peritoneal cytology	Positive	17/34 (50%)	10/34 (29%)	0.1364
	Negative	17/34 (50%)	24/34 (71%)	
Operation	Complete	30/34 (88%)	31/35 (89%)	1
	Non‐complete	4/34 (12%)	4/35 (11%)	

**TABLE 5:** Univariate and multivariate analyses of overall survival in ovarian clear cell carcinoma patients

Clinicopathological parameteres						
Parameter	Univariate			Multivariate		
	Hazard Ratio	95% CI	*p*‐value	Hazard Ratio	95% CI	*p*‐value
Age	1.68	0.35–6.38	0.4779			
Stage	4.55	1.12–17.34	0.0354	4.54	1.08–17.95	0.0394
(1–2 vs 3–4)						
Peritoneal cytology	2	0.53–8.08	0.3003			
Lymph node metastasis	3.64	0.76–14.02	0.0988			
Operation	5.66	1.19–21.66	0.0321			
Inhibin‐α	9.06	1.66–168.17	0.0078	9.03	1.63–168.61	0.0086

**TABLE 6:** Univariate and multivariate analyses of disease‐free survival in ovarian clear cell carcinoma patients

Clinicopathological parameteres						
Parameter	Univariate			Multivariate		
	Hazard Ratio	95% CI	*p*‐value	Hazard Ratio	95% CI	*p*‐value
Age	1.88	0.64–4.99	0.2358			
Stage	2.73	0.93–7.21	0.0649	2.69	0.92–7.17	0.07
(1–2 vs 3–4)						
Peritoneal cytology	2.61	1.00–7.20	0.0499			
Lymph node metastasis	2.44	0.77–6.61	0.1201			
Operation	2.2	0.51–6.75	0.2581			
Inhibin‐α	6.02	1.96–26.13	0.0011	6.02	1.95–26.26	0.0011

## 4 DISCUSSION

**FIGURE 9:**
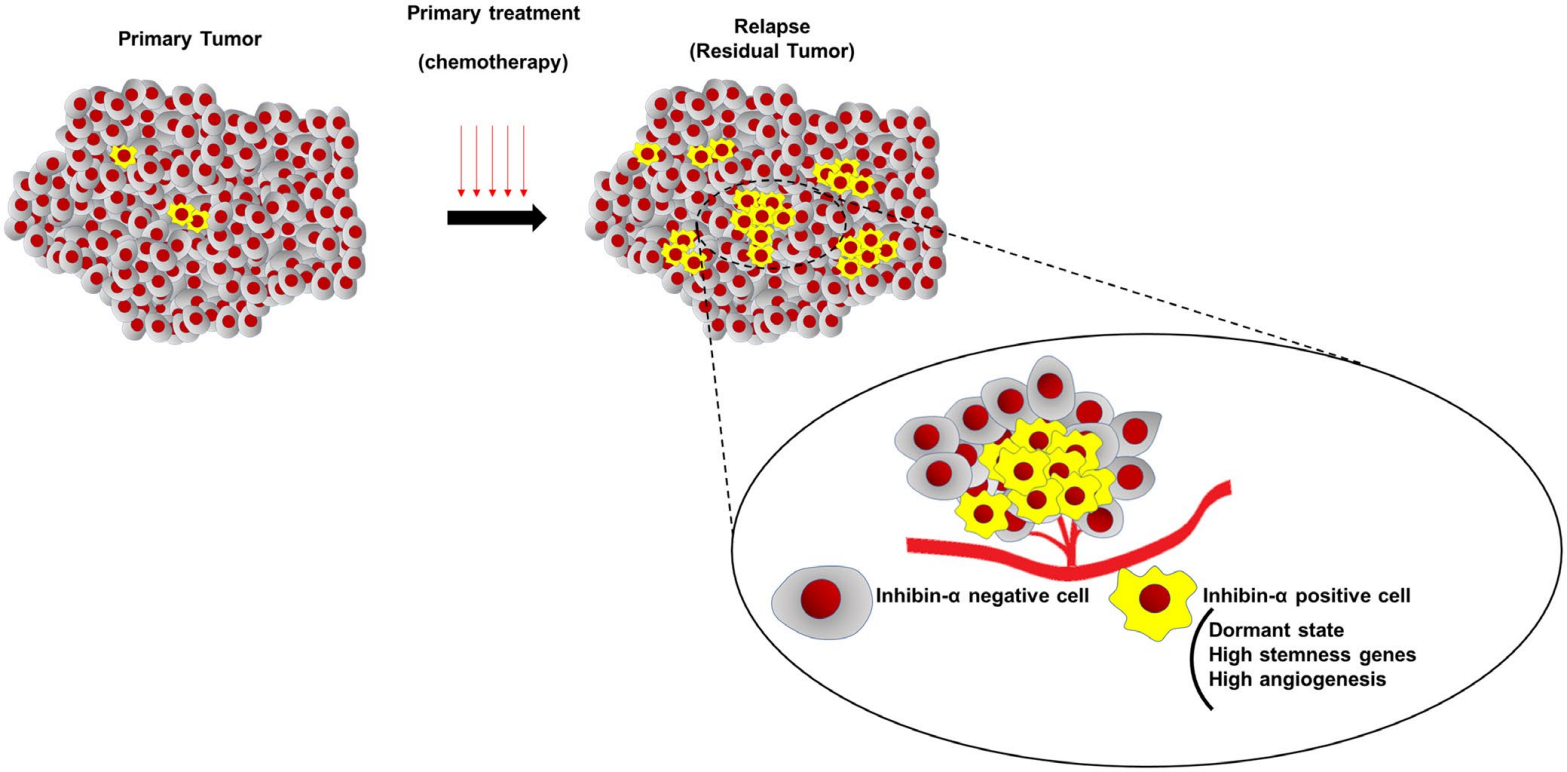
Scheme of OCCC heterogeneity based on inhibin‐α‐positive cells. There are some cases with inhibin‐α‐expressing tumor cells, which are less proliferative and immature minor subpopulation with angiogenic potential. This subpopulation may be responsible for resistance against anticancer treatment, such as chemotherapy and radiotherapy

In this study, we identified that inhibin‐α was expressed in less proliferative tumor epithelial cells of OCCC and upregulated in the recurrent site, spheroids and chemoresistant cells (Figure 9). Moreover, we showed inhibin‐α‐positive cells had immature properties and high angiogenic potential, leading to unfavorable prognosis (Figure 8). These findings suggested that inhibin‐α would be not only a prognostic biomarker but also a biomarker for immature and treatment‐resistant cancer cells.

Tumor dormancy plays a key role in cancer relapse.^12^ Inhibin‐α was expressed in the less proliferative tumor tissues. Of the three OCCC cell lines, we used OVISE, which formed a lot of spheroids, to compare 2D and 3D cultures. Most of the 2D cultured cells were not in the G0 phase cell cycle, and their ALDH1A1 and inhibin‐α expression was low. On the other hand, most of the 3D cultured cells were in the G0 phase cell cycle, and their ALDH1A1 and inhibin‐α expression was higher than in 2D cultured cells. Chemotherapy has been associated with an increase in immature tumor cells.^25^, ^26^, ^27^, ^28^, ^29^ We created chemoresistant cells using RMGV and OVTOKO cell lines whose spheroids were hardly formed. In both chemoresistant cells, significant number of spheroids were observed, and ALDH1A1 and inhibin‐α expression level increased compared to that of parent cells. As for clinical samples with recurrent OCCC, inhibin‐α‐positive cells increased in recurrent lesions compared to those in the initial lesions. Furthermore, immature subpopulation of tumors, called cancer initiating/stem cells, is reported to be related with the VEGF family, and especially VEGF‐A is important for their maintenance.^30^, ^31^, ^32^, ^33^, ^34^ Inhibin‐α‐positive cells showed much higher VEGF‐A and VEGF‐C expression than inhibin‐α‐negative cells. Considering these results, inhibin‐α‐positive cells would possess similar features to cancer initiating/stem cells in OCCC.

When comparing the OS and DFS rate of 69 patients, inhibin‐α‐positive cases showed unfavorable prognosis and a higher recurrence rate. Nonetheless, there was a major limitation in this study that could be addressed in future research. The number of OCCC patient samples was particularly small. Although this was taken care of in our statistical analysis and the results of the statistical analyses certainly suggested the important roles of inhibin‐α in prognosis of OCCC patients, further investigation based on a larger cohort would be desirable. These findings were consistent with the above‐mentioned results that inhibin‐α‐positive cells reflect immature and treatment‐resistant cancer cells. The appearance of those cells would increase the possibility of metastasis/recurrence and have a significant impact on recurrence risk and prognosis. Although stage and surgery completion rate are considered to be important prognostic factors,^35^, ^36^, ^37^, ^38^, ^39^ inhibin‐α expression was a prognostic factor independent of these existing items. Inhibin‐α could be an important new information for deciding treatment strategy.

In conclusion, we have identified the prognostic value of inhibin‐α‐expressing cells, which were less proliferative and immature. Our results may help to define new strategies for treating OCCC.

## CONFLICT OF INTEREST

The authors declare that they have no conflict of interest.
